# Phacoemulsification cataract surgery: what you need to know

**Published:** 2023-12-01

**Authors:** William Dean, Rengaraj Venkatesh

**Affiliations:** 1 Assistant Clinical Professor: ICEH, LSHTM, UK. Honorary Associate Professor: University of Cape Town, South Africa.; 2 Consultant: Speciality Director, Gloucestershire Hospitals NHS Foundation Trust, UK.; 3 Chief Medical Officer: Aravind Eye Hospital, Pondicherry, India.

**The need for phaco is increasing – especially for those patients with less mature cataracts.**

**Figure:**
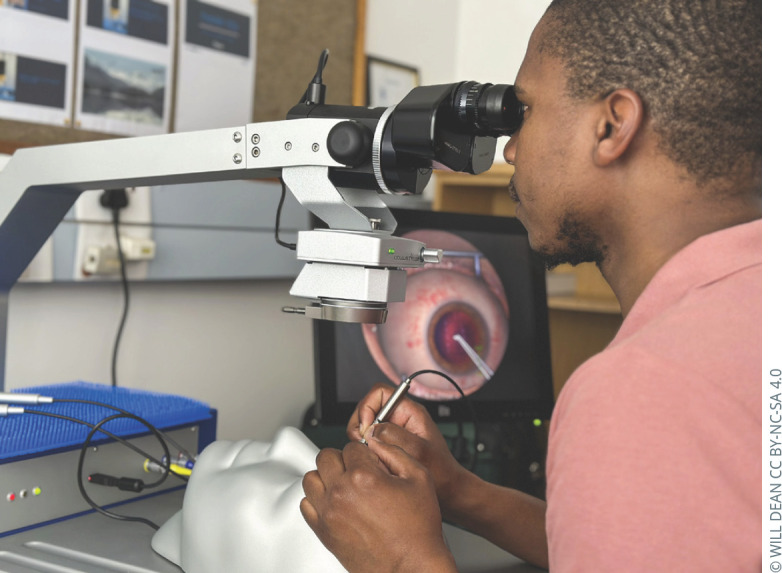
Phaco training using a high-fidelity simulator. **SOUTH AFRICA**

Setting up a phacoemulsification (phaco) cataract service from the ground up can be daunting, as it requires suitable equipment and preparation. Ensure your facility is prepared to handle phaco surgery with properly maintained equipment, adequate supplies of phaco cassettes and consumables, ophthalmic viscosurgical devices (OVD), specialised phaco instruments, and an adequate microscope with an excellent coaxial illumination source.

There are also several considerations beyond the training of surgeons and teams. Biometry will be essential, along with a reliable supply of foldable IOLs across a whole range of powers. Supplies are more expensive than those needed for manual small-incision cataract surgery (MSICS), and these will need to be budgeted for. The equipment will need regular maintenance, and personnel will need to know how to troubleshoot in case of problems. A voltage stabiliser and uninterruptable power supply will be needed if electrical supply is unreliable at your facility. Is there vitreo retinal support available from surgeons in the area, in case of a dropped nucleus? Speak to colleagues for advice.

**From the field**

**The need for phaco**

**Hillary Rono** is an ophthalmologist working in Kenya's Ministry of Health. He is also the Country Director of Peek Vision in Kenya.

“In Kenya, patients are increasingly demanding phacoemulsification (phaco) cataract surgery, partially due to its reputation for more rapid healing and better visual outcomes.

“Preliminary results from the Rapid Assessment of Avoidable Blindness (RAAB) survey study in Kenya show that existing cataract surgical services (using the Manual Small-Incision Cataract Surgery, or MSICS, technique) are reaching those who are blind or have severe visual impairment due to cataract. However, people with mild or moderate visual impairment due to cataract are not offered MSICS surgery, due to the risks associated with MSICS procedures in patients with less mature cataracts.

“To address this gap, there is a need for more surgeons to learn phaco surgery. It is important that surgeons retain their MSICS training, however, as it will be needed at various times in their practice, including when they encounter difficulties during phaco surgery.”

## How to practice skills for phaco using simulation

Learn the basics of phacodynamics and fluidics by reading standard manuals and visiting websites (listed below in ‘Useful training resources’). Remember that there is a steep learning curve. Learn the feel of the phaco foot pedal while listening to the sounds of the machine in various pedal positions and sampling other functions you may need. Familiarise yourself with the settings on the machine (phaco power, vacuum, aspiration rate).

**Before** operating on live patients, surgeons learning phaco must undergo simulation training (on animal or artificial eyes) in as realistic an operating theatre environment as possible. Practice finger positioning on the large handpiece, hand positioning to manage the weight of the handpiece, and foot pedal activation until you can control the handpiece easily and intuitively.

Virtual reality (VR) simulation training in phacoemulsification is also possible using the training modules available with the Eyesi^®^ surgical simulator, which has been shown to reduce complication rates in operations performed by trainee surgeons by up to 38%.^1^ The Eyesi^®^ is especially useful for practicing capsulorrhexis. Any virtual reality or other simulation training must always be supported by live surgical observation as well as supervised practice.

## Useful training resources

The PGY2, PGY3 and PGY4 Residents’ videos on cataractcoarch.com are an excellent resouce:

https://cataractcoach.com/2020/01/25/list-of-key-videos-for-residents/

Orbis International's Cybersight website has a ‘Fundamentals of Phacoemulsification’ course:

https://cybersight.org/online-learning/

Sullivan P, Benjamin L, Little B. Phacoemulsification Surgery: An Interactive Multimedia Atlas for Ophthalmology Trainees. The PDF version is available from https://bit.ly/46CBYjM and an interactive version from the iBooks store.

Simulatedocularsurgery.com offers videos for practicing phaco cataract surgery: https://simulatedocularsurgery.com/cataracts

*A longer version of this article is available at* www.cehjournal.org and on our app.
